# Genome-wide identification and analysis of the invertase gene family in tobacco (*Nicotiana tabacum*) reveals NtNINV10 participating the sugar metabolism

**DOI:** 10.3389/fpls.2023.1164296

**Published:** 2023-06-02

**Authors:** Lingtong Cheng, Jingjing Jin, Xinxi He, Zhaopeng Luo, Zhong Wang, Jun Yang, Xin Xu

**Affiliations:** ^1^ China Tobacco Gene Research Center, Zhengzhou Tobacco Research Institute of CNTC, Zhengzhou, China; ^2^ Technology Center, China Tobacco Hunan Industrial Co., Ltd., Changsha, China

**Keywords:** invertase, tobacco, expression pattern, plant growth and development, 3D model

## Abstract

Sucrose (Suc) is directly associated with plant growth and development as well as tolerance to various stresses. Invertase (INV) enzymes played important role in sucrose metabolism by irreversibly catalyzing Suc degradation. However, genome-wide identification and function of individual members of the INV gene family in *Nicotiana tabacum* have not been conducted. In this report, 36 non-redundant NtINV family members were identified in *Nicotiana tabacum* including 20 alkaline/neutral INV genes (*NtNINV1-20*), 4 vacuolar INV genes (*NtVINV1-4*), and 12 cell wall INV isoforms (*NtCWINV1-12*). A comprehensive analysis based on the biochemical characteristics, the exon-intron structures, the chromosomal location and the evolutionary analysis revealed the conservation and the divergence of NtINVs. For the evolution of the NtINV gene, fragment duplication and purification selection were major factors. Besides, our analysis revealed that NtINV could be regulated by miRNAs and cis-regulatory elements of transcription factors associated with multiple stress responses. In addition, 3D structure analysis has provided evidence for the differentiation between the NINV and VINV. The expression patterns in diverse tissues and under various stresses were investigated, and qRT-PCR experiments were conducted to confirm the expression patterns. Results revealed that changes in *NtNINV10* expression level were induced by leaf development, drought and salinity stresses. Further examination revealed that the NtNINV10-GFP fusion protein was located in the cell membrane. Furthermore, inhibition of the expression of *NtNINV10* gene decreased the glucose and fructose in tobacco leaves. Overall, we have identified possible *NtINV* genes functioned in leaf development and tolerance to environmental stresses in tobacco. These findings provide a better understanding of the *NtINV* gene family and establish the basis for future research.

## Introduction

Invertase (INV) is called sucrose or β-fructofuranosidase (EC 3.2.1.26), which can catalyze the hydrolysis of sucrose to produce fructose and glucose, and is a key enzyme in sucrose metabolism (Sturm, 1999), playing an important physiological role in plants (Sherson et al., 2003). Invertase is not only present in photosynthetic tissues but also in non-photosynthetic tissues, which is an important enzyme in plant carbohydrate metabolism and involved in plant growth and development, organ building, stress response, etc. According to the optimal PH, invertases could be classified into acid invertases (AINV) and neutral invertases (alkaline invertases) (NINV). Furthermore, it could also be divided into three categories depending on their location in the cell. NINVs are usually localized in chloroplasts, mitochondria or cytoplasm and belong to the glycosidase 100 (GH100), whereas AINVs could be located in the cell wall (CWINV) or vacuole (VINV) and belong to the glycosidase 32 (GH32) (Veillet et al., 2016; Zhu et al., 2021).

It is generally accepted that AINV originates from aerobic bacteria and eukaryotes, and NINV from cyanobacteria (Vargas et al., 2003; Koch, 2004). Of these, CWINV and VINV, both are AINVs, have an acidic pH optimum and NINVs usually have the alkaline or neutral pH optimum. NINVs can only hydrolyze sucrose owing to lacking the N-terminal signal peptide. By comparison, AINV has two conserved motifs, the β-fructosidase motif (NDPD/NG) and the cysteine catalytic domain motif (WECV/PD). Therefore, AINVs can catalyze some oligosaccharides, such as cottonseed, in addition to sucrose. However, it also shows differences among the AINV two groups. VINVs have a valine/isoleucine residue in the catalytic “WEC-P/V-D” box, while CWINVs feature a proline residue. In general, *NINV* is extremely unstable and is difficult to purify which caused little known about its structure and VINV has been characterized with two sheets of six β-strands which are called the β-Sandwich Module (Alberto et al., 2004).

The INV family genes have been identified in many plants, such as *Arabidopsis* (Ji et al., 2005), rice (Ji et al., 2005), bamboo (Zhu et al., 2021), maize (Juárez-Colunga et al., 2018) and tomato (Ahiakpa et al., 2021). The function and regulatory mechanism of INV genes have also been documented. INV has been shown to play a role in a wide range of physiological and developmental processes, including sucrose partitioning (Sherson et al., 2003), seed and pollen development (Chourey et al., 2006), flower and fruit development (Roitsch and González, 2004; Yu et al., 2008), shoot and root growth (Martín et al., 2013) and environmental responses(Proels and Hückelhoven, 2014; Liu et al., 2015). For example, after decreasing the levels of AINV in muskmelon, the sucrose concentration in the fruit increased and the fruit size decreased in transgenic plants (Yu et al., 2008). Down-regulation of the *Ta-A/N-Inv1* increased the disease resistance to *Puccinia striiformis* of wheat by increasing cytoplasmic hexose accumulation (Liu et al., 2015). *Nin88* played important role in tobacco during pollen development (Engelke et al., 2010; Le Roy et al., 2013). Knockdown of vacuolar invertase gene in potato resulted in a decrease in post-harvest cold-storage sugars formation in potatoes (Yasmeen et al., 2022). *AtCYT-INV1* regulated drought stress-induced inhibition on lateral root growth by controlling hexose concentration in Arabidopsis cells (Qi et al., 2007). *MeNINV1*-overexpressing Arabidopsis had higher A/N-INV activity, the increased glucose, fructose content in the leaves as well as promoted plant growth and delayed flowering time (Wang et al., 2022). The expression pattern of the INV gene were influenced by a number of factors, such as plant hormones, biotic stresses, abiotic stresses, and sugar signaling (Proels and Hückelhoven, 2014; Wang et al., 2014; Veillet et al., 2016). INVs also showed various expression patterns between different tissues and developmental periods of the plant. In addition, invertase inhibitors also regulated the expression of INVs and influence the plant physiological activity (Xu et al., 2017).

Tobacco is an important economic crop in the world. Sucrose is usually synthesized in tobacco leaves *via* photosynthesis and transferred to the other tissues to maintain the normal activity of tobacco plant and response to environmental stresses. INV is critical in the sucrose metabolism pathway in tobacco and several researches have been reported. For example, the *cwINV* regulates the acquisition of carbohydrates to support the hypersensitive reaction during the plant suffering the pathogens (Essmann et al., 2008). Over-expression of the yeast-derived invertase in tobacco showed abnormal growth accompanied by reducing root formation, as well as starch and soluble sugars accumulation in leaves (Sonnewald et al., 1991). However, research about the INV gene of tobacco is still scarce, and the INV gene family remains unexplored at a genome-wide level in tobacco. In this research, a comprehensive analysis of INV gene members in tobacco was conducted, including gene structure, conserved motifs, sequence phylogeny, gene synteny, 3D structure, and expression patterns in various tissues and stresses. We also built a regulation network for NtINVs, transcript factors (TFs), and miRNAs. This study aimed to: (1) identify the INV members in *Nicotiana tabacum*; (2) analyze the expression patterns of *NtINV*s under various stresses and tissues; and (3) analyze the potential function of the *NtINV*s in tobacco plants.

## Materials and methods

### Identification of the INV gene family in the tobacco

For the identification of INV genes in tobacco, the protein sequence of 17 Arabidopsis INV members as queries were searched in Sol Genomics Network (Fernandez-Pozo et al., 2015) and China tobacco genome database using the TBLASTN (v2.12.0) (McGinnis and Madden, 2004). The candidate tobacco INV genes were predicted and further confirmed using the HMMER (v3.3.2) (Finn et al., 2011), PFAM (http://pfam.xfam.org/) and SMART (http://smart.embl-heidelberg.de/) based on the INV conserved domain (NINV: PF12899, CWINV: PF08244 and PF00251, and VINV: PF11837, PF08244, and PF00251). And molecular weight (MW) and isoelectric points (pI) of INV proteins were predicted by online ExPASy tool (http://web.expasy.org/protparam/) (Gasteiger et al., 2003). Signal peptide sequence and potential cleavage site of NtINVs were conducted by SignalP 5.0 program (Almagro Armenteros et al., 2019). The amino acid sequence of INV proteins was analyzed by the online tool TMHMM server v2.0 program (http://www.cbs.dtu.dk/services/TMHMM/).

### 3D structure prediction

The 3D (3-dimensional) structure of the protein is important for understanding detail function mechanism. 3D structure of NtINV proteins were predicted by AlphaFold2 (Jumper et al., 2021), and displayed by Pymol software (http://pymol.org/). Molecular docking was predicted by Autodock_vina (Trott and Olson, 2012).

### Phylogenetic, gene structure and conserved motif analysis

The phylogenetic tree of the *NtINV* genes was constructed using the neighbour-joining (NJ) method with a bootstrap value of 1000 using MEGA X (Kumar et al., 2018). The gene structures of each *NtINV* gene was conducted by TBtools (v1.09867) (Chen et al., 2020). The motifs were analyzed using the MEME program (http://meme-suite.org/tools/meme), and the identified motifs were annotated by InterProScan (http://www.ebi.ac.uk/Tools/pfa/iprscan/), and the optimum width of motifs was set to 6–200 amino acid residues, and the maximum number of motifs was set to 20, and the other settings were kept default values.

### Chromosomal location and gene duplication analyses

For gene duplication analysis, amino acid sequences of NtINVs were aligned with Blast (v2.12.0, e-value = 1e-5). The tandemly duplicated and segmental duplicated (collinearity) analysis of NtINVs using the MCScanX software with the default settings. The non-synonymous (*Ka*) and synonymous substitution (*Ks*) rates of duplicated NtINV genes, and the ratio of Ka/Ks were calculated using the ParaAT2.0 program to evaluate the selection pressure (Zhang et al., 2012). And the identified NtINVs distributed on chromosome was visualization by using the TBtools.

### Promoter analysis and miRNA–*NtINV* interaction prediction

The 1, 500 bp upstream sequence from the transcription start site of *NtINV* genes was used to analyse the cis-elements using PlantCARE(http://bioinformatics.psb.ugent.be/webtools/plantcare/html/). The relationship between NtINVs and transcription factor were retrieved from plant transcription factor database PlantTFDB (http://planttfdb.cbi.pku.edu.cn). And tobacco miRNAs were obtained from the miRBase database (Kozomara et al., 2019). The miRNA sites for *NtINV*s were searched by PsRNATarget (http://plantgrn.noble.org/psRNATarget/) with default settings. The interaction networks were visualization by Cytoscape (Smoot et al., 2011).

### Expression pattern analysis

All the filtered high-quality reads were mapped to the tobacco reference genome using HISAT2 (v2.2.1) (Kim et al., 2019) with default parameters. StringTie (v2.1.7) (Pertea et al., 2015) and FeatureCounts (v1.6.4) were used to obtain read counts for all annotated genes in the tobacco genome. Transcripts per million (TPM) was used to measure the expression level. The Raw RNA-seq data about cold (Jin et al., 2017), drought (Yang et al., 2017a), cadmium (He et al., 2016), topping (Chen et al., 2019), ABA (Wu et al., 2021), CMV (Liu et al., 2019) and P.nicotianae (Yang et al., 2017b) were obtained from SRA database (Leinonen et al., 2011). Transcriptome data about 8 distinct tissues including leaf, vein, blade, stem, root, axillary bud, callus, and seed of tobacco were used to investigate the expression patterns of *NtINV* genes and the raw sequence data of the tissues were download from PLncDB (http://plncdb.tobaccodb.org/) (Jin et al., 2021). The sampling stages for each tissue are listed in **Supplementary Table S6**.

### Experimental materials

The cultivated tobacco variety K326 (*N. tabacum*) was used in the analysis of the expression of INV genes in various tissues and stress treatment. The seedlings were cultivated in plastic pots with a 16 h light photoperiod at 28°C during the day and at 23°C at night. The plant root, stem, leaf, axillary bud, and flower samples were collected, as described in our previously study (Wang et al., 2015). For cold stress, salinity stress, drought stress, sucrose, and various plant hormone treatments were according to the previous researches (Xie et al., 2021). Untreated plantlets were applied as control (CK). The treated and control plantlets were collected 6 hours after treatment and then all the samples were immediately frozen in liquid nitrogen and stored at −80°C. Leaves at 6 different maturity groups were collected at the same time and defined as M1, M2, M3, M4, M5 and M6. Middle leaves (MLs) at the 3rd to 5th positions were selected for this study. The stages of maturities were judged by the previous study (Ougham et al., 2008). Each sample, comprising three uniformly grown plants, was analyzed in triplicate under the same conditions. All collected samples were quickly frozen in liquid nitrogen and stored in a refrigerator at -80°C.

### RNA isolation and quantitative real-time RT-PCR analysis

Total RNA of tobacco tissues was extracted with an RNA Kit (Imagene, Beijing, China). DNA contamination was removed by digestion with RNase-free DNase I (Takara, Beijing, China). Reverse Transcriptase M-MLV (Takara) was employed to synthesize first-strand cDNA using 1 μg of total RNA as a template. qRT-PCR was applied using an SYBR Green kit (Imagene) in a 20 μl reaction solution. The PCR program was as follows: 95°C for 30 s, 40 cycles of 95°C for 10 s, 60°C for 30 s. The expression levels of the target genes were standardized to the expression level of the *NtGAPDH* gene using the 2^−△△^Ct method. Three independent biological replicates were performed for each gene. The primers used for qRT-PCR analysis are listed in **Supplementary Table S8**.

### Subcellular localization analysis of NtNINV10

The coding sequence of NtNINV10 excluding the termination codon was amplified by Phanta Max Master Mix (Vazyme, Nanjing, China). Then, these sequences and green fluorescent protein (GFP) fragments were inserted into the pC1300-GFP vector, which was driven by the CaMV35S promoter. The reconstructed vectors were confirmed by sequencing. The recombinant constructs and control were transiently transformed and expressed in Arabidopsis protoplasts. Then, the GFP fluorescence signals were captured using a confocal microscope (FV 1200 OLYMPUS, Japan).

### Virus-induced NbibenNINV silencing

To elucidate the biological functions of tobacco INV, *NbibenNINV10* was selected for gene silencing. The fragment and the empty pTRV2 (PYY13) vector were digested separately using PstI restriction enzymes. Then, the fragment was ligated into digested pTRV2 vectors, and confirmed by sequencing. Thus construct was obtained, namely TRV-NINV10. The vectors were then transferred into Agrobacterium tumefaciens strain GV3101 using freeze-thaw method. The tumefaciens strains containing TRV-NINV10 were grown at 28 °C in Luria Bertani (LB) medium containing appropriate antibiotics. The cells were harvested and resuspended in the infiltration buffer (10 mm MES, pH = 5.5, 200 μm acetosyringone, and 10 mM MgCl2) to a final absorbance (optical density (OD) at 600 nm) of 1.0 and incubated for 2 h at 25 ± 2 °C. For leaf infiltration, each A. tumefaciens strain containing TRV-NINV10 and pTRV1 were mixed in a 1:1 ratio in infiltration buffer and infiltrated into lower leaves using a 1 ml needleless syringe. The empty pTRV2 vector and its derivative, TRV-PDS construct were used as negative and positive controls, respectively, using the same method.

### Determination of sugar content in leaves

The sugar content of fresh tobacco leaves was determined using the Sucrose-glucose-fructose content kit (enzymatic method) (Geruisi, Suzhou, China). Briefly, sucrose was hydrolyzed to fructose and glucose under the acidic conditions, and the product had a characteristic absorption peak at 620 nm, which was used to calculate the sucrose content. Fructose was converted to glucose by specific enzymes, glucose in the presence of an enzyme complex such as hexokinase to produce NADPH. The contents of sucrose, glucose and fructose are calculated by detecting the increase of NADPH at 340nm. Each independent VIGS lines were performed with three replicates.

## Results

### Genome-wide identification of INVs in tobacco

Based on the 17 INVs from *Arabidopsis*, we used BLAST and HMMER to search for the *NtINVs* against the tobacco genome. Finally, a total of 36 non-redundant *NtINVs* were identified, which included 20 NINVs, 12 CWINVs, and 4 VINVs (**Supplementary Table S1**). The protein length of NtINVs proteins ranges from 506 (*NtCWINV9*) to 687 (*NtCWINV7*) amino acid residues, with an average of 326 amino acids (**Supplementary Table S1**). The relative molecular weight varies from 58.03 (*NtCWINV9*) to 77.05 kDa (*NtCWINV7*), whereas the isoelectric points range from 5.22 (*NtCWINV1*) to 9.37 (*NtCWINV9*). Subcellular analysis showed that the majority of NtNINV proteins (18/20 = 90%) were localized on the chloroplast, and all of them lacked the N-terminal signal protein. By contrast, majority of *NtVINV*s are localized in the vacuole and cytoplasm (**Supplementary Table S1**). It may indicate the different physiological functions between the *NtNINV* and *NtVINV*.

### Phylogenetic, gene structure and conserved motif analysis of *NtINV* gene family

**Figure 1:**
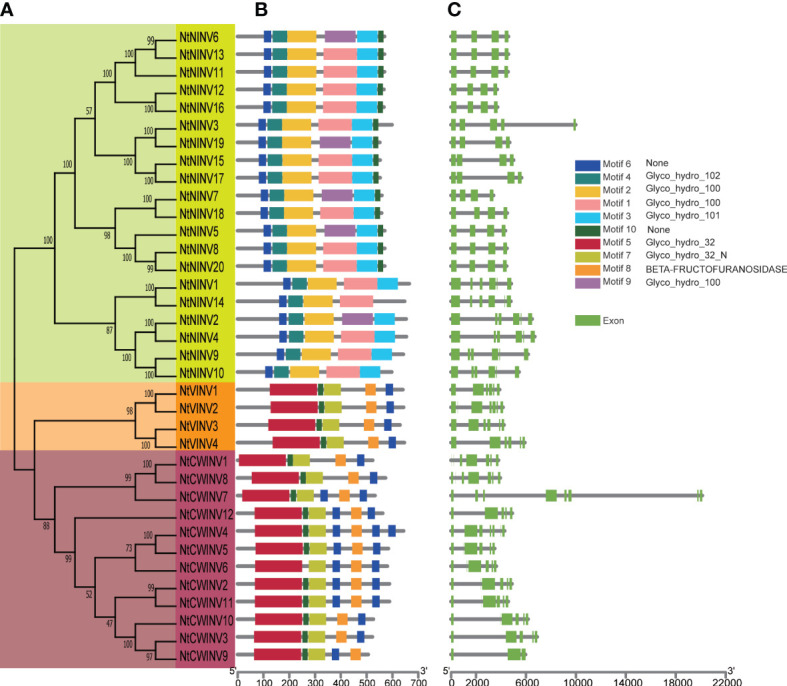
Phylogenetic tree, conserved motif and gene structure of *NtINV* genes. **(A)** Phylogenetic relationship among the *NtINV* genes based on the amino acid sequence alignment. **(B)** Conserved motifs in amino acid sequence of different sub-groups **(I-III)** of *NtINV*s. The 10 different colors of the boxes on the right represents diverse conserved motifs. **(C)** Exon-intron analysis of 3 subgroups of *NtINV*s.

The phylogenetic analyses revealed that *NtINV* genes could be divided into 3 subgroups (**Figures 1A**, **S1**). The large subgroup NINV consisted of 20 *NtNINV* members, whereas the small subgroup VINV contained 4 *NtVINV* members. Subgroup CWINV is composed of 12 *NtINV* members. Next, the 10 most conserved motifs for *NtINV*s were explored by the MEME program and annotated by InterProScan. Three motifs (1, 2, and 9) were annotated as Glyco_hydro_100, which were present in most of *NtNINV*s (75%, 100%, and 25%) (**Figure 1B**; **Supplementary Table S2**), which suggested the motif 2 had been preserved for a long time in the NINV of tobacco. Interesting, all members in subgroup VINV and CWINV have motif 4, 5, 7, and 8, which were annotated as Glyco_hydro_102, Glyco_hydro_32, Glyco_hydro_32N, and BETA-FRUCTOFURANOSIDASE, respectively. These common domains indicated the similar functions for these two subgroups. As for the unannotated motif 6 and 10, motif 10 was only found in some NtNINVs, whereas motif 6 was identified in all NtINVs. Moreover, some CWINVs might have several motif 6, which suggested it may play important roles in CWINV family.

To obtain further insights into the genetic structure of INV evolution, we diagrammed the exon-intron structures of *NtINV* genes. The results demonstrated structural variation among these *NtINV* genes, ranging from 3 to 8 exons, whereas most *NtNINV*s contained 4 exons and 3 introns, and *NtCWINV*s and *NtVINV*s contained most genes with 6 exons and 5 introns (**Figure 1C**). Furthermore, 65.0% (13/20) of *NtNINV* genes had 4 exons, all *NtVINV*s had 6 exons, and 41.7% (5/12) of *NtCWINV*s had 6 exons (**Figure 1C**). Generally, *NtINV* genes in the same subgroup exhibit similar exon-intron characteristics, consistent with their phylogenetic relationship.

### Distribution and duplication of *NtINV* family

**Figure 2:**
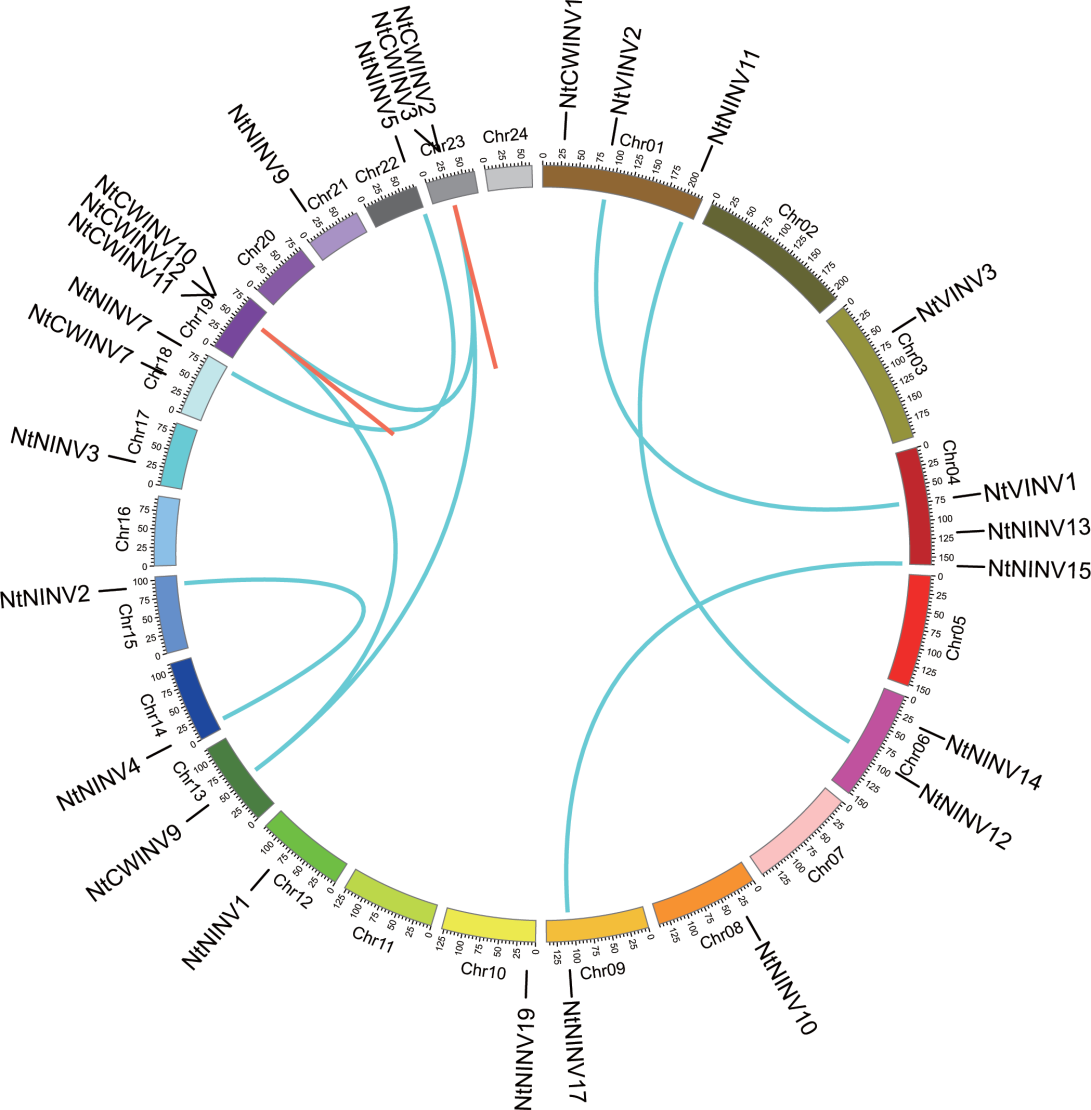
Distribution of tandem and segmental duplicated INV genes in the tobacco genome generated by Circos. Tandem and segmental duplicated gene pairs are linked by the red in same chromosome and cyan lines between chromosomes, respectively.

To understand the distribution of NtINV, physical map was constructed, as shown in **Figure 2**. Totally, 26 *NtINV*s could be mapped on the tobacco chromosomes, and 10 genes could not be mapped on any of the chromosomes but mapped on certain scaffolds. There were 3 *NtINV* genes on each of the chromosomes 1, 4 and 19, 2 *NtINV* genes on each of the chromosomes 6, 18 and 23, and only 1 gene on chromosome 3, 8, 9, 10, 12, 13, 14, 15, 17, 21 and 22. Notably, we observed that some genes from the same subfamily formed gene clusters, such as the CWINV cluster on Chr19 and Chr23 (**Figure 2**; **Supplementary Table S3**). We inferred that gene clusters may be generated from tandem duplications and segmental duplications. It was well known that segmental and tandem duplications were two major mechanisms for gene family expansion in plants. Thus, *NtINV* gene pairs were evaluated using MCScanX. As a result, a total of 18 duplication events consisting of 24 paralogs were identified, including 3 tandem duplications and 15 segmental duplications. Results suggested segmental duplications were the major contribution to the evolution and expansion of the *NtINV* family. In general, the nonsynonymous (*Ka*) and synonymous (*Ks*) ratio was used to evaluate the driving force underlying the gene evolution. *Ka*/*Ks* > 1 means a positive selection, *Ka*/*Ks <*1 means a negative selection, and *Ka*/*Ks* = 1 means a neutral selection. Therefore, the ratio of *Ka*/*Ks* was calculated to evaluate evolutionary selection for the INV duplication gene pairs. The results showed that *Ka*/*Ks* values of *NtINV* duplicate genes ranged from 0.0617 to 0.2530, indicating that the INV family in tobacco suffered strong negative selection during the evolution process.

### Cis-acting elements in the promoter region of *NtINV*s

The upstream promoter region of the genes possesses many cis-acting elements which could regulate the gene expression. To better understand the potential regulatory mechanisms of *NtINV* genes, we tried to identify the presence of cis-elements in the promoter regions of *NtINV* genes. The identified cis- elements were further classified into 4 distinct groups based on their putative functions (**Figure S2**; **Supplementary Table S4**). Regulatory elements related to abiotic or biotic stress were found to be the largest in number and comprise 13 cis-elements, including cold, light, anoxic, anaerobic, drought, wounds, and defenses and stress responsive elements. A total of 291 genes were involved in light response elements (LTRs) (**Figure S2**; **Supplementary Table S3**). There are seven classes of elements associated with hormone responses, with the largest number of classes associated with abscisic acid (ABA), followed by methyl jasmonate (MeJA), gibberellin (GA), salicylic acid (SA) and growth hormone, respectively (**Figure S2**; **Supplementary Table S4**). In addition, there were five classes of elements that regulated plant development, including fenestrated leaf sarcomeres, endosperm, seeds and meristem-specific expressed elements. These results suggest that the NtINV family may be involved in a complex network of stress responses and hormonal regulation, as well as plant growth and development.

### 3D structure prediction of NtINV protein

**Figure 3:**
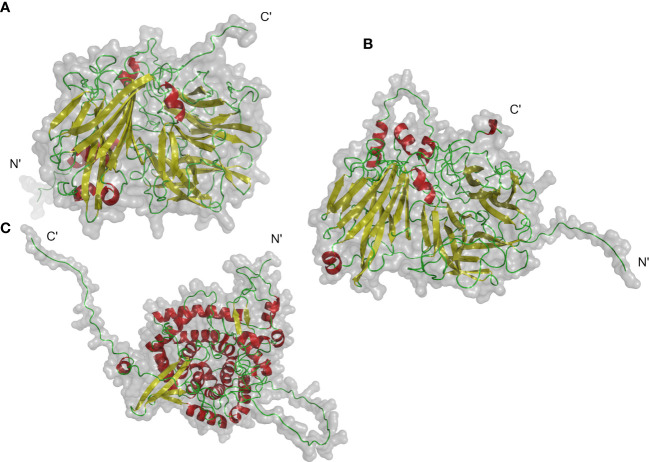
Putative 3D structures of 3 category invertase proteins of tobacco. **(A)** *NtCWINV2* (acid invertase), **(B)** *NtVINV1* (acid invertase), **(C)** *NtNINV5* (neutral invertase). Colors indicate secondary structures: α-helix (red), β-sheets (yellow), loops (green).

The protein 3D structure provides useful information for mechanistic understanding of INV function. Previous analysis has shown that *NtCWINV2* contained a putative signal peptide with a predicted vacuole location, whereas *NtNINV5* and *NtVINV1* did not have the putative signal peptide with a predicted chloroplast and cytoplasm location, respectively (**Supplementary Table S1**). Hence, we tried to predict the 3D structure of these INVs using AlphaFold2 (**Figure 3**). The ERRAT test scores by SEVER server for 3D models (*NtCWINV2*, *NtVINV1*, and *NtNINV5*) were 89.21, 87.99, and 95.32, which indicated that predicted 3D models were reliable. Our results showed that these three proteins were monomers that contained β-sheet and α-helices. All the three proteins had characteristic loops at their N-terminals, but only *NtVINV1* had the α-helices at the C-terminals. Similarly, both *NtCWINV2* and *NtVINV1* had more β-sheet than the α-helices, whereas the *NtNINV5* had more α-helices than the β-sheet (**Figure 3**). These results may imply that the *NINV* and *VINV* have different mechanisms.

### Regulation networks for *NtINV*s

Our studies have revealed that hormone-responsive elements are enriched in the promoter regions of *NtINV* genes. We speculated that the corresponding transcription factor (TF) might directly regulate *NtINV* genes. Hence, we tried to explore the regulatory relationship between transcription factors and *NtINV*s using PlantTFDB. In total, 1,214 TFs members from 33 families might play important roles in the regulation of *NtINV*s (**Figure S3**; **Supplementary Figures S3**–**S6** and **Supplementary Table S5**). Many TF families involved in plant organ development were identified, including MIKC_MADS, TCP, and BBR-BPC, et al. And there are also many TF families implicated in stress responses were found, such as ERF, WRKY, MYB, HD-ZIP, NAC, and Dof, et al. Among them, a total of 224 ERF transcription factors, including the AP2 and RAV subfamily, were most abundant. Furthermore, we also investigated potential miRNA binding sites for *NtINV*s using PsRNATarget. Finally, 38 miRNA families consisting of 93 miRNAs may have regulatory relationships with *NtINV*s (**Supplementary Figures S2**–**S4** and **Supplementary Table S5**). Most miRNAs had several *NtINV* targets, including nta-miR827 which could target 10 *NtINV* genes (**Figure S3**; **Supplementary Table S5**). By contrast, *NtCWINV7* could be targeted by 37 miRNAs, including nta-miR156 and nta-miR171 (**Figure S2**; **Supplementary Table S5**). However, further research into the regulation of *NtINV*s and TF/miRNA is required.

### Expression analysis of *NtINV* genes in eight representative tissues

**Figure 4:**
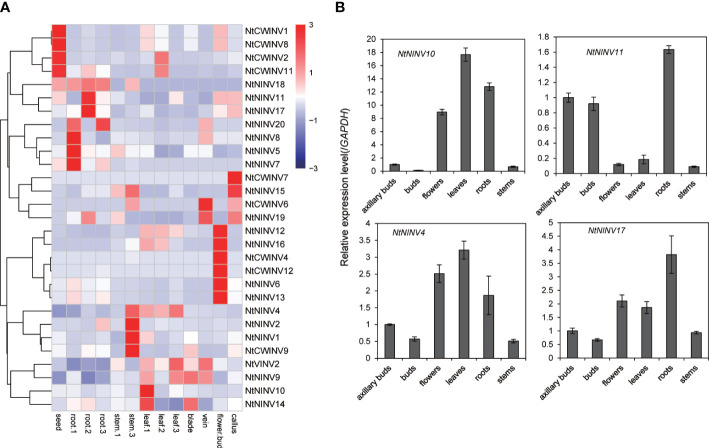
Expression profiles of tissues-specific *NtINV* genes in different tissues. **(A)** Heatmap of the expression in different tissues at three development stages [seedling (1), mature (2), and two days after topping (3)], scaled by rows. **(B)** Results of qRT-PCR analysis of four selected *NtINV*s in axillary bud, flower, leaf, root, and stem.

To investigate the expression patterns of *NtINVs* in different tissues, eight tissues were used to analyze the expression of *NtINVs*. As shown in **Figure 4A**, many NtINVs exhibited different tissue-specific expression patterns. In addition to 7 *NtINVs*, 29 genes were expressed in at least one tissue (**Figure 4**; **Supplementary Table S6**). It’s worth noting that several genes that belong to the same subfamily have similar tissue expression patterns. Results showed that some *NtCWINV* genes were highly expressed in seeds, suggesting that these CWINV genes might play important functions in tobacco seed development, such as *NtCWINV1*, *NtCWINV2*, *NtCWINV8* and *NtCWINV11*. Interestingly, several *NtNINV* genes were highly expressed in root tissues, revealing their significant roles in root growth, such as *NtNINV11*, *NtNINV17*, *NtNINV18*, and *NtNINV20*. Furthermore, *NtCWINV1* might play an important role in callus differentiation due to it’s highly expression in callus. These results were similar to those from qRT-PCR analyses (**Figure 4B**). *NtNINV11* and *NtNINV17* showed higher relative transcription levels in roots, respectively. Additionally, *NtNINV4* and *NtNINV10* showed higher relative transcription levels in leaves, respectively (**Figure 4B**). Consistent with phylogenetic and motif analysis, the specific and various expression profiles of *NtINV* genes in different tissues also suggested their diverse roles. Interesting, we also found that some duplicated gene pairs displayed similar expression patterns among these 13 tissues, like the *NtNINV5* and *NtNINV7*, *NtNINV12* and *NtNINV16*, *NtNINV8* and *NtNINV20*, which may indicate their similar functions (**Figure 4A**).

Moreover, expression patterns of *NtINV*s also varied in three development stages (seedling, mature, and two days after topping stages). For the genes expressed in roots, 4 (*NtNINV*5, *7*, *8* and *20*) showed relatively stronger expression during seedling stages, and *NtNINV11* and *NtNINV17* were strongly expressed during mature stages, suggesting that these genes might play some specific roles during early stages or mature stages of root development. For the genes expressed in leaves, some INV genes displayed high expression levels in the seedling stage, like the *NtNINV14*, and some INVs were highly expressed at the maturing stage (*NtCWINV2*) or two days after the topping stage (*NtVINV2*), which indicated their different roles in the leaf’s different development stages.

**Figure 5:**
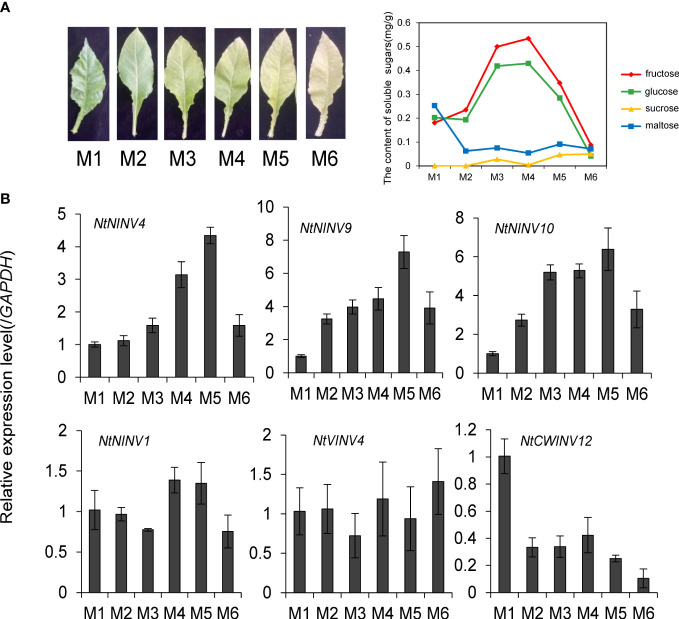
Expression patterns of 6 *NtINV* genes in tobacco leaves at six leaf development stages. **(A)** The phenotype and sugar content of tobacco leaves at different leaf development stages. **(B)** The qRT-PCR results of 6 *NtINV* genes in tobacco leaves at different leaf development stages.

To understand the INV gene function during tobacco leaf development and senescence, we collected the tobacco leaves from six maturity stages (M1-M6) to analyse the expression changes of some *NtINV*s. The content of chlorophyll in tobacco leaves is gradually decreasing from M1 to M6 (**Figure S8**), and the content of glucose and fructose in leaves first increased and then decreased, reaching the highest at M3 and M4 stage (**Figure 5A**). To clarify the INV gene expression, we conduct the qRT-PCR experiment in the 6 different stage leaf tissues, and esults showed *NtNINV4*, and *9*genes showed a gradual increase in expression as the leaves matured, with the highest expression level at the M5 stage, and *NtNINV10* has relatively high expression at M3-M5 stage (**Figure 5**). When the leaves totally become yellow and senescence, their expression decreases. These results implied the potential roles of the *NtNINV*s in tobacco leaf development and maturing.

### Expression analysis of *NtINV* genes in response to various abiotic and biotic stresses

**Figure 6:**
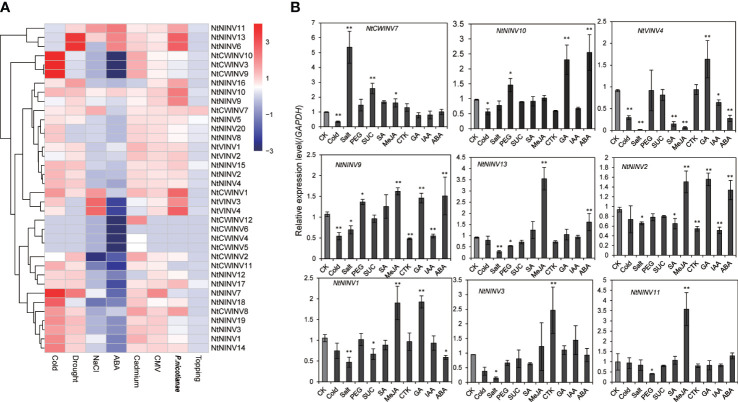
Expression patterns of *NtINV* genes under various stress treatments. **(A)** Expression changes of *NtINV* genes under cold, ABA, NaCl, cadmium, topping, *P. nicotiana*, drought, and CMV stresses. The expression change is indicated by the ratio of FPKM value of the treatment to that of the control (CK). **(B)** Results of qRT-PCR analysis of 9 *NtINV* genes in response to stresses (cold, salt, sucrose, and drought) and hormones (GA, CTK, SA, JA, IAA, ABA) treatment. * indicate P < 0.05, ** indicate P < 0.01.

To further explore the responses of *NtINV*s during various stress responses, the expression patterns of all *NtINV*s were investigated using publically available transcriptome data. As shown in **Figure 6A**; **Supplementary Table S7**, many down-regulated *NtINV* genes were identified under the ABA treatment, and only 7 genes were up-regulated. Moreover, more INV genes were up-regulated under the cold treatment and drought treatment than down-regulated, whereas more INVs were down-regulated under the salt treatment. Results showed *NtINV* genes were extremely sensitive to cold stress and exhibited extremely down-regulation or up-regulation, such as *NtCWINV9/10* increased more than 64 times compared to control. Additionally, three *CWINV*s were up-regulated under the cadmium treatment, suggesting their potential roles in the cadmium response. Only a few *NtINV* genes changed slightly after the topping treatment. Among various biotic stresses, *NtINV*s showed significant responses upon inoculation with *Phytophthora nicotianae* (*P. nicotianae*) and *Potato Virus Y* (PVY), whereas only a few genes just slightly changed under *cucumber mosaic virus* (CMV) treatment. It is interesting that most *NtINV*s are specifically involved in individual stress treatment, rather than universal response. Beside, we also found that some duplicated gene pairs displayed similar expression patterns under eight treatments, including the *NtNINV4* and *NtNINV2*, *NtCWINV9* and *NtCWINV10*, *NtVINV3* and *NtVINV4*.

To confirm the expression pattern results of *INV* genes in response to abiotic stress and compare the transcription patterns of *NtINV* genes between different hormone treatments (Gibberellins (GA), methyl jasmonate (MeJA), salicylic acid (SA), abscisic acid (ABA), cytokinin (CTK), indole-3-acetic acid (IAA)), qRT-PCR was further conducted to investigate the expression patterns (**Figure 6B**). Similar with RNA-seq analysis, some *NtINV* genes were extremely sensitive to cold stress and exhibited significant down-regulation, such as *NtNINV9*, *NtCWINV7*, and *NtVINV4*. Some genes showed a significant up-regulation under salt or drought, like the *NtCWINV7* under 200 Mm NaCl treatment and the *NtNINV10* under 20% PEG treatment. Moreover, *NtCWINV7* was also significantly up-regulated under 1% sucrose. Additionally, the expression level of *NtNINV1* and *NtVINV4* was significantly reduced under ABA treatment, whereas *NtNINV2*, *NtNINV9*, *NtNINV10*, and *NtNINV13* appeared to be up-regulated. *NtNINV1*, *NtNINV11*, and *NtNINV13* were significantly up-regulated under the MeJA treatment. Some INVs, such as *NtNINV1*, *NtNINV2*, *NtVINV4*, *NtNINV9*, and *NtNINV10* were also up-regulated in response to GA treatment. However, IAA and CTK treatment induced the expression of *NtNINV2* and *NtNINV9* slightly, and *NtNINV3* was significantly increased under CTK. *NtNINV2* and *NtVINV4* were down-regulated under the SA. In summary, these results demonstrate that the expression profiles of *NtINV* genes varied under different abiotic and biotic stresses.

It was noted that *NtNINV10*, a highly expressed gene in the leaves, and its expression changes with leaf maturity. It also showed the greatest increase in expression level under cold and drought treatment. The expression changes of the *NtNINV10* genes indicated that it might play important roles in regulating the sugar content to response the stress and regulating the leaf development. Therefore, *NtNINV10* was selected to detect its function in participated the sugar metabolism.

### Subcellular localization of *NtNINV10*

**Figure 7:**
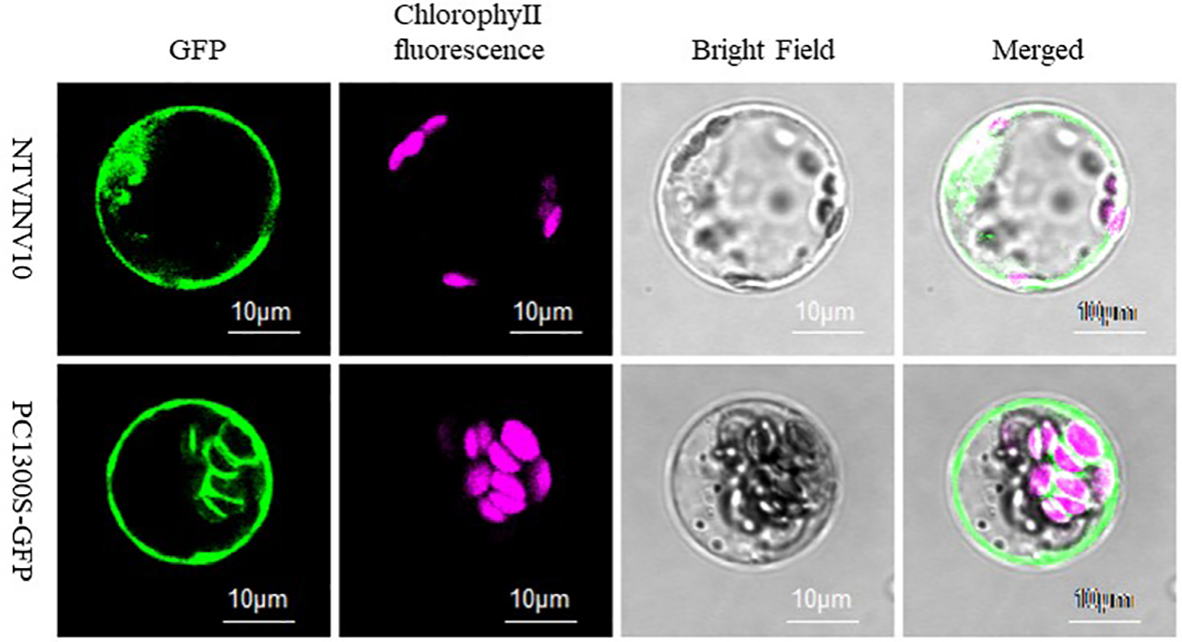
Subcellular localization of NtNINV10. Confocal images were captured the day after agroinfiltration. NtNINV10-GFP represents the gene and GFP represents the control with an empty vector.

The NtNINV10 protein was predicted to be localized in the chloroplast by Plant-mPLoc and iPSORT, while it was predicted to be localized in the cytoplasm by WoLFPSORT. To clarify the *NtNINV10* subcellular localization, *NtNINV10* fused with GFP was transformed in Agrobacterium and inoculated in in Arabidopsis protoplasts. As shown in **Figures 7**, **S9**, the NtNINV10 protein was localized to the plasma membrane. This result indicated the NtNINV10 mainly existence in the plasma membrane.

### Soluble sugar content changes in NbibenNINV10-silenced plants

**Figure 8:**
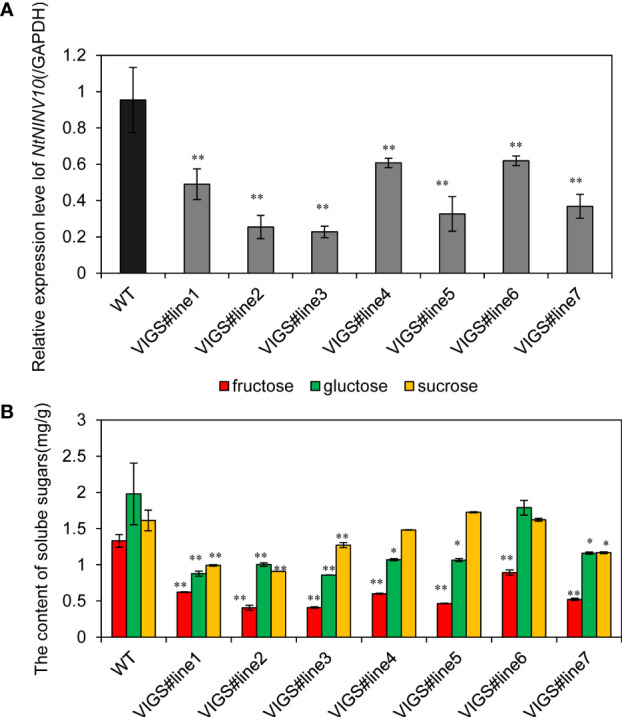
Soluble sugar content changes in virus-mediated NINV gene silencing in N. benthamiana. **(A)** The relative expression levels of *NbibenNINV10* in different plants. **(B)** The soluble sugar content difference between TRV- *NbibenNINV10* and negative control plants. Columns and bars represent the means and standard erorrs (n = 3), respectively.*indicate P < 0.05, **indicate P < 0.01.

Soluble sugar have been long proven to play important roles in plant growth and development, and to further investigate the function of tobacco INV genes, we generated virus-induced gene silencing constructs, namely TRV-NINV10. The expression of *NbibenNINV10* in silenced plants decreased to 35.11%~76.13% of the negative control plant. Next, we measured glucose, fructose and sucrose content (**Figure 8**) of the VIGS lines. Compared with the negative controls, the contents of fructose was decreased in TRV-NINV10 plants, with decrements of 33.08% ~68.47%, and the contents of glucose was reduced by 41.35%~ 56.62%.

## Discussion

Invertases play important roles in plant growth and development, and they participate in the sucrose metabolism, signal transduction, and stress responses to plant growth (Ahiakpa et al., 2021). Our study is the first comprehensive and systematic report for the characterization of the *NtINV* gene family in *N. tabacum*. Many INV families have been identified in many plants, but the number of INV families varies greatly among different plants. A total of 36 INV genes were identified in the tobacco genome. This number is larger than that of most reported species, such as *Arabidopsis* (17) (Ji et al., 2005), rice (19) (Ji et al., 2005), tea (14) (Qian et al., 2016), bamboo (29) (Zhu et al., 2021), maize (21) (Juárez-Colunga et al., 2018) and tomato (24) (Ahiakpa et al., 2021). This indicates the considerable expansion of the INV gene family in tobacco compared to other plant species. In plants, gene family expansion is usually the result of species polyploidy and gene duplication. The expansion of the gene family primarily occurs *via* three modes: segmental duplication of multiple genes, tandem duplication of individual genes, and whole-genome duplication (Panchy et al., 2016). Gene duplication ultimately culminates in producing proteins with sub-functionalization, neo-functionalization, or non-functionalization (Glasauer and Neuhauss, 2014). We have identified 18 duplication events involving 24 paralogs. Segmental duplication is thought to be the main driver of *NtINV* evolution, as 83.3% (15) of these are segmental duplications (**Supplementary Table S3**). *Ka* and *Ks* analyses revealed that the evolution of INV genes was mainly through purifying selection. Nevertheless, all 18 duplicated gene pairs had *Ka*/*Ks* < 1 (**Supplementary Table 3**), indicating that *NtINV*s had undergone negative selection pressures with limited functional divergence after duplication. We also found some duplicated genes showed similar expression patterns between different tissues or under different treatments, which may reflect their similar function during evolution.

In previous research, INV members could be clustered into AINV and structurally unrelated NINV based on the similarity of protein sequence and biochemical property of their pH optima. AINV proteins are disturbed into cell wall invertase (CWINV) or vacuole invertase (VINV) according to their subcellular localization (Liu et al., 2016). A/NINV proteins are diverged into several distinct isoforms localized in the cell membrane, cytoplasm, nucleus, chloroplast, and mitochondria (Liu et al., 2015; Ahiakpa et al., 2021). Unlike CWINVs and VINVs that act at acidic pH of 4.5-5.5, NINVs function at an optimal pH of 7.0 -7.8, which usually lack the N-terminal signal peptide. Our analysis illustrated that the phytogenic relationship, the intron-exon structure, and the protein motif distribution of *NtINV* genes were strongly associated. According to the phylogenetic relationships, the NtINV family was categorized into 3 subgroups, and the VINV and CWINV subgroups belong to the AINV. In this study, 27.8% of *NtINVs* have been considered as membrane-binding proteins because their N-terminus contains transmembrane domains, which indicate the secretory nature of these INVs (**Supplementary Table S1**). Moreover, members of the *NtNINV* subfamily were predicted to be localized in the cytoplasm and chloroplasts, and all of them lacked N-terminal signal peptides, which were similar with previous research (Sturm, 1999). The protein sequence analysis showed that NtAINVs and NtNINVs possessed different functional domains at their C-terminus, including the GH32 domain and the GH100 domain, respectively, and shared one short non-functional domain motif 6. The difference was that motif 6 existed once in the N-terminal of NtNINV, whereas motif 6 was in the C-terminal of NtVINV, and existed more than once in the CWINV subgroup. Moreover, the N-terminus of INVs having similar signal peptides for subcellular localization belonged to the same subgroups. Therefore, we speculated the particular function of unannotated motif 6 for *NtCWNINV* groups, and it may also influenced the subcellular differentiation of the 3 subgroups, which needed further research to understand its function. Interestingly, the motif 10 was only identified in the one branch of *NtNINV* subgroup, and it is consistent with the previous research, which NINV could be classified into α and β branches (Zhu et al., 2021).

Previous study has revealed that the C-terminal residues (from 306 to 432) of *T. maritima* invertase have two sheets of six β-strands which are called the β-Sandwich Module. This module functions as a carbohydrate recognition domain in the formation of higher oligomers (Alberto et al., 2004). The 3D structures of 3 INVs were predicted and visualized from ab initio, respectively. The N-terminal structure was diversity between them, which may explain their different subcellular localization. NINV and VINV member have different structural features, which indicate probable different mechanisms, and it is also consistent with the functional diversity and optimal pH of these two different enzymes. Their predicted 3D structure also revealed that *NtINV*s contained two sheets of six β-strands (**Figure 3**). These data may support the idea that the β-Sandwich Module helps plant VINV to recognize and catalyse sucrose and other β-fructoses, including oligosaccharides, whereas the NINV only specifically catalyse sucrose.

In plants, transcription factors (TFs), regulatory RNAs and enzymes form a complex gene regulatory network (GRN) that regulates plant growth and development, and stress response (Yamaguchi-Shinozaki and Shinozaki, 2005; Ibraheem et al., 2010; Wang et al., 2021). Analysis of cis-elements in the promoter of the NtINV gene led to the detection of four main types of cis-elements associated with biotic/abiotic stress, hormone responses, developmental processes and other transcription-related elements.

Analysis of the cis-elements in *NtINV* gene promoters resulted in the detection of four major types of cis-elements associated with biotic/abiotic stress, hormone response, developmental processes and other transcription related elements. Therefore, these findings have provided some basic evidence that the INV genes are important in the sucrose metabolism, various plant processes and stress responses. A relatively large number of light-responsive cis-elements were detected in *NtINV* promoters, revealing that *NtINV*s might participate in the light response. In addition, a series of cis-elements related to abiotic stress responses and plant development were identified in INV gene promoters, such as MYB, ERF, MIKC_MADS, and TCP, which may regulate gene expression under various stresses and during plant development. Hence, the INV regulation network constructed in our study might help to better understand plant sucrose metabolism in response to stress and in plant growth and development.

Tissue expression analysis revealed that *NtINV* family genes are widely expressed in the roots, stems, leaves, blades, veins, axillary buds, calluses, and seeds. And many genes were tissue-specifically expressed, suggesting their specific role in different tissues or developmental stages. As shown in **Figure 4** a majority of highly expressed *NtCWINV* may play important roles in tobacco seed development, and a majority of highly expressed *NtNINV* in tobacco roots, suggested their potential roles in tobacco root development. Furthermore, many *NtCWINV*s and *NtNINV*s were found highly expressed in axillary buds, implying their important roles in tobacco axillary buds development. Similar results have been reported in previous research, which has confirmed that *NINV* plays a critical role in Arabidopsis roots (Sergeeva et al., 2006). The CWINV gene could also regulate seed development in rice and tomatoes (Wang et al., 2008; Jin et al., 2009). Extracellular invertase was exhibited in high amounts in the tuberized lateral branches of transgenic tobacco lines (Guivarc’h et al., 2002).

To understand the potential roles of *NtINV* genes in stress resistance, public RNA-seq data was used to investigate their expression patterns. The complexity and diversity of *NtINV* expression patterns under various biotic or abiotic stresses were observed. In tobacco, the expression levels of the majority of *NtINV* genes decreased after drought, salt, and *P. nicotianae* infection treatment, while the expression levels of many members of the INV family increased after cold and cadmium treatment. This suggested that INV has different mechanisms for responding to various stresses (**Supplementary Figure S7**). Meanwhile, individual INV genes are usually sensitive to one specific external stress, and some INV genes are widely responsive to various biotic and abiotic stresses. For example, *NtCWINV12* was the only response under cadmium stress. *NtNINV10* was located in the chloroplast and was associated with drought and cold stress. Plant hormones such as ABA, ethylene, SA, and MeJA are considered to be involved in plant response to various stresses and accelerate plant senescence (Khan et al., 2013; Yu et al., 2019). Here, we selected significantly response NtINV members to further explore under various plant hormone treatments by qRT-PCR. Salt and sucrose treatments resulted in increased transcript levels of *NtCWINV7*, and *NtCWINV7* also increased under the MeJA treatment, possibly indicating *NtCWINV7 via* the MeJA and sugar signaling pathway in tobacco to adapt to salt stress. This is consistent with the MeJA and sugar signal function in alleviating the salt stress in plants (Yu et al., 2019). The expression level of *NtNINV10* was increased under drought stress, GA and ABA, and *NtNINV10* also showed a high transcript level at the tobacco leaf yellowing stage. Meanwhile, silencing *NtNINV10* displayed the decreased fructose and glucose content, which implied its potential role in sucrose metabolism, leaf development and drought stress responses. *NtINV*s responded to different hormones and stress by displaying different expression patterns, possibly indicating the functional diversity of INV gene family members. These results provide useful insights into the potential capabilities of *NtINV*s involved in plant growth and development, and various stresses.

## Conclusions

In this study, we have identified and functionally analyzed members of the INV gene family in tobacco by conducting phylogeny, protein property, 3D models and expression patterns analysis. Many INV genes are tissue-specific expressed, and some of these genes may be involved in some specific biotic and abiotic stresses. We found the expression NtNINV10 is related to leaf maturity, and also regulated by ABA, drought, etc. Furthermore, silencing the expression *NtNINV10* influenced the sugar metabolism especially the glucose and fructose content. Overall, our work provides a solid foundation for understanding the function of the INV gene in leaf maturing and stress responsiveness by participate in plant sugar metabolism.

## Data availability statement

The datasets presented in this study can be found in online repositories. The names of the repository/repositories and accession number(s) can be found in the article/**Supplementary Material**.

## Author contributions

XX and JJ conceived and designed the research; LC performed bioinformatics data analysis; XH, ZW, ZL and JY sampled the materials and performed the experiments; XX and JJ wrote the manuscript. All authors contributed to the article and approved the submitted version.

## Supplementary material

Supplementary Figure S1 Phylogenetic analysis of invertase (INV) families. The Neighbour-Joining (NJ) phylogenetic tree was constructed according to amino acid sequences of INV genes in Arabidopsis, rice, tobacco the functionally evaluated invertase gene from cucumber, carrot, wheat, tomato, maize and potato by MEGA 7.0. The INV proteins were divided into three groups, distinguished by different colors. The standard value of nodes was derived using bootstrapping, with 1000 replicates and the functionally evaluated invertase gene were marked with the triangle.

Supplementary Figure S2 Frequency distribution characteristics of cis-acting regulatory elements (biotic/abiotic stress, hormone response, and development group) in the promoter sequences of *NtINV* genes.

Supplementary Figure S3 Regulation network of *NtINV*s in tobacco. The green, pink and purple boxes represent *NtINV* genes, TFs and miRNAs, respectively. Grey line represents the interaction.

Supplementary Figure S4 Regulation network of *NtCWINV*s in tobacco.

Supplementary Figure S5 Regulation network of *NtNINV*s in tobacco.

Supplementary Figure S6 Regulation network of *NtVINV*s in tobacco.

Supplementary Figure S7 Expression patterns of *NtGAPDH* under various stress treatments or in different tissues.

Supplementary Figure S8 The chlorophy content of tobacco leaves at different leaf development stages.

Supplementary Figure S9 Subcellular localization of NtNINV10 into the leaf of *Nicotiana benthamiana*.

Supplementary Figure S10 The diverse functions and roles of *NtINV*s.
