# Supplementary material for: Genome-wide identification and analysis of the invertase gene family in tobacco (Nicotiana tabacum) reveals NtNINV10 participating the sugar metabolism
Source: Front Plant Sci. 2023 Jun 2;14:1164296. doi: 10.3389/fpls.2023.1164296 (PMC10272776; doi:10.3389/fpls.2023.1164296)
Supplement: Supplementary Figure S1 — Phylogenetic analysis of invertase (INV) families. The Neighbour-Joining (NJ) phylogenetic tree was constructed according to amino acid sequences of INV genes in Arabidopsis, rice, tobacco the functionally evaluated invertase gene from cucumber, carrot, wheat, tomato, maize and potato by MEGA 7.0. The INV proteins were divided into three groups, distinguished by different colors. The standard value of nodes was derived using bootstrapping, with 1000 replicates and the functionally evaluated invertase gene were marked with the triangle. [file Presentation_1.pptx]

## Slide 1
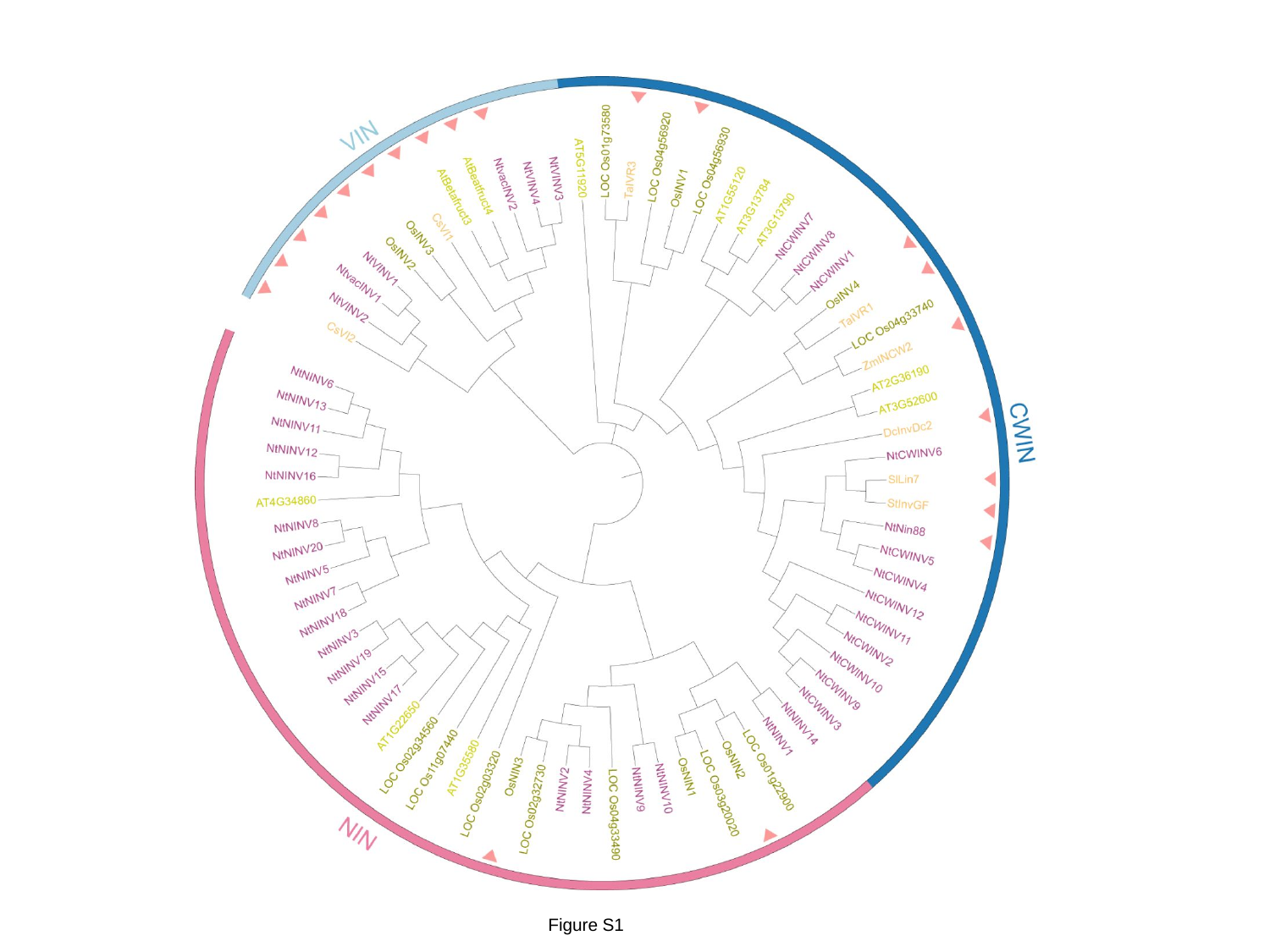

Figure S1

## Slide 2
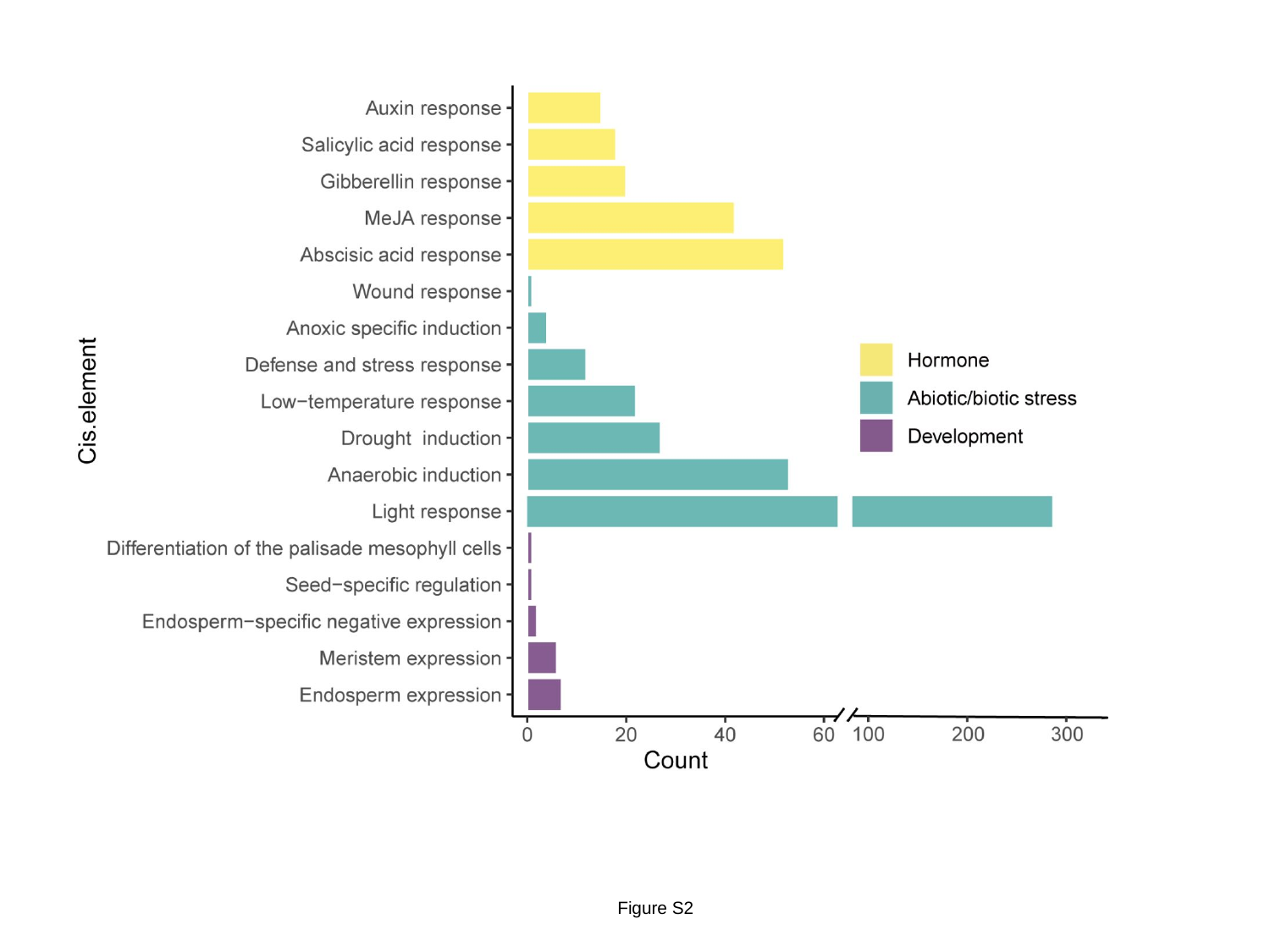

Figure S2

## Slide 3
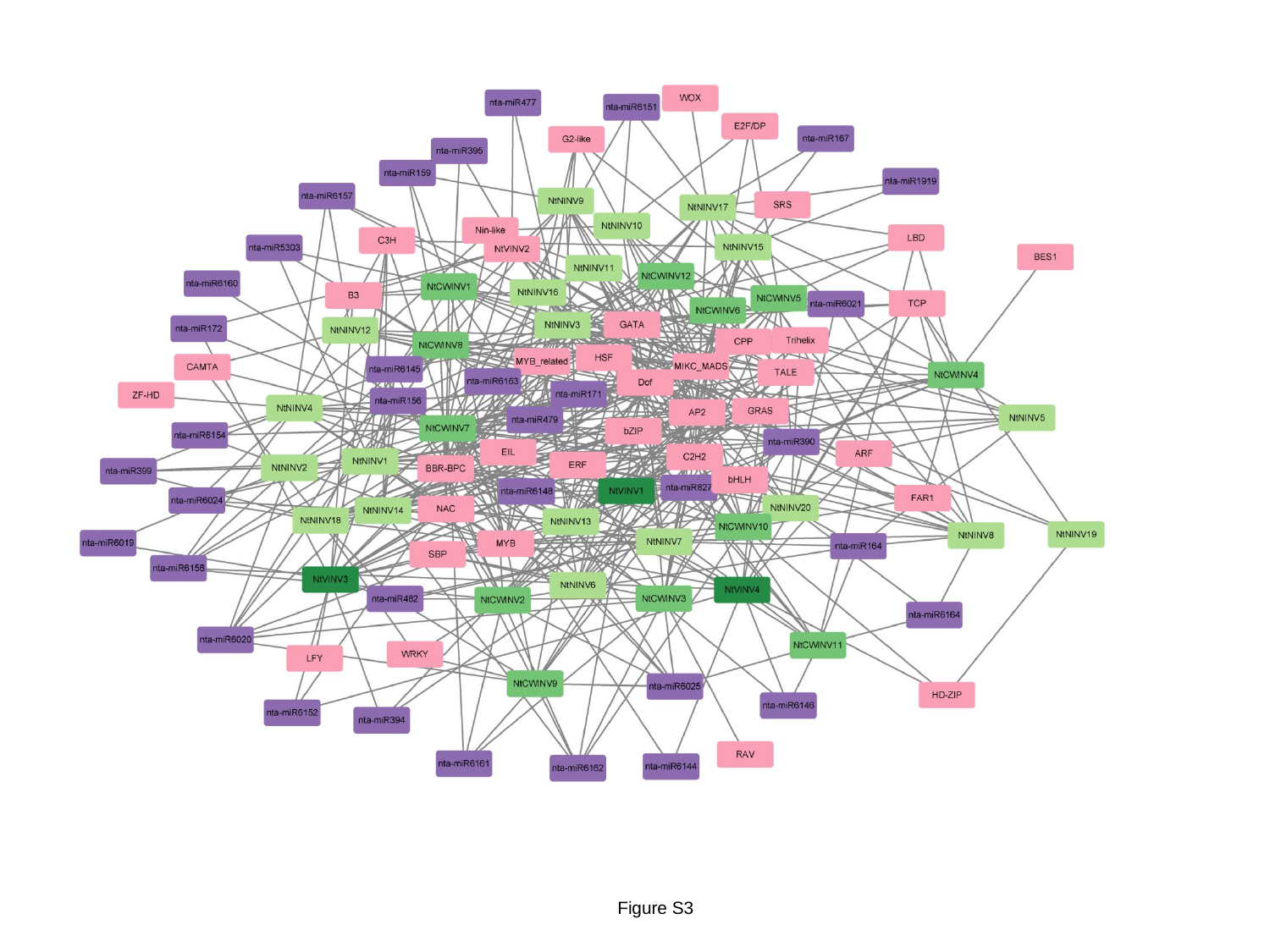

Figure S3

## Slide 4
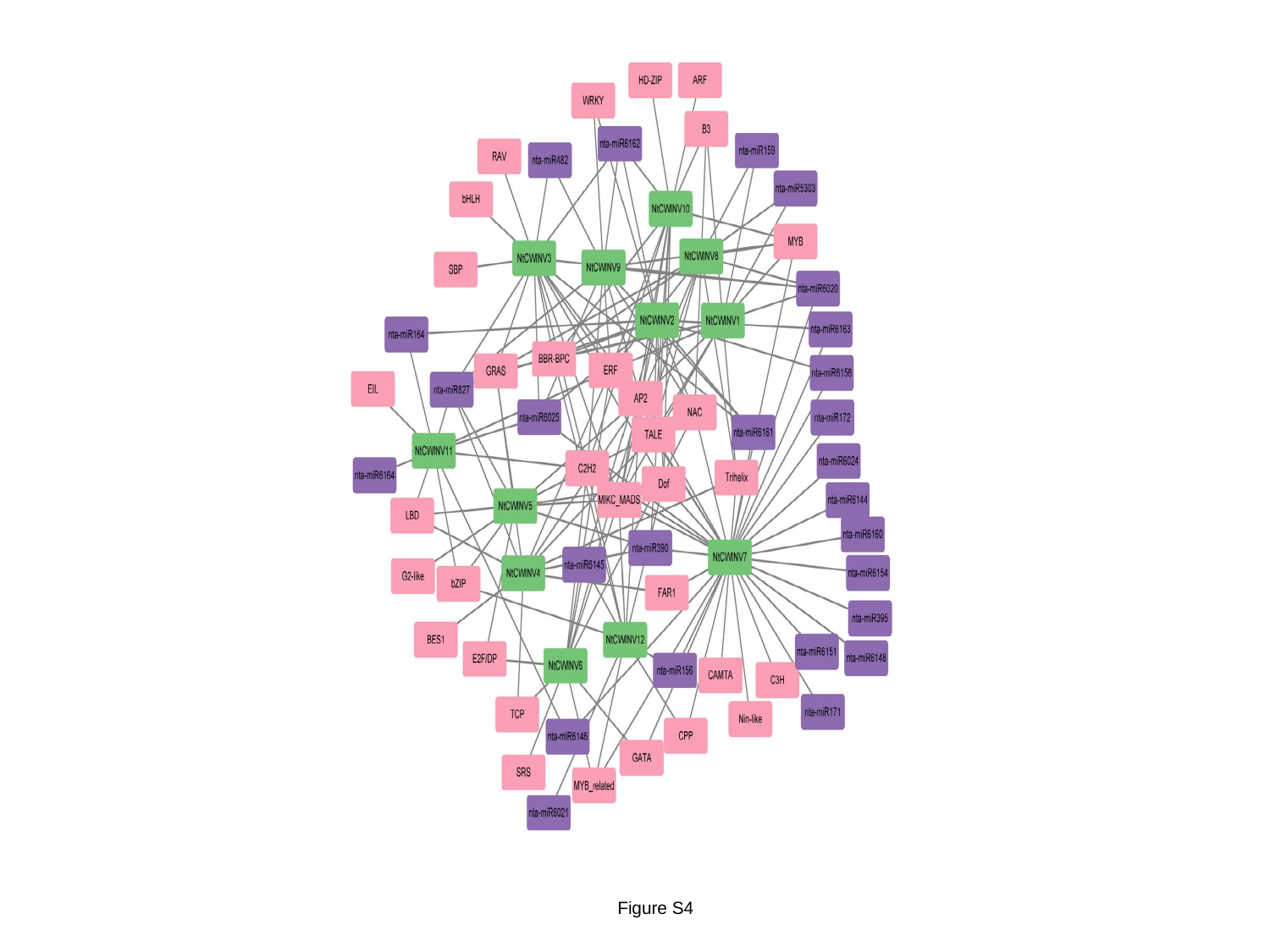

Figure S4

## Slide 5
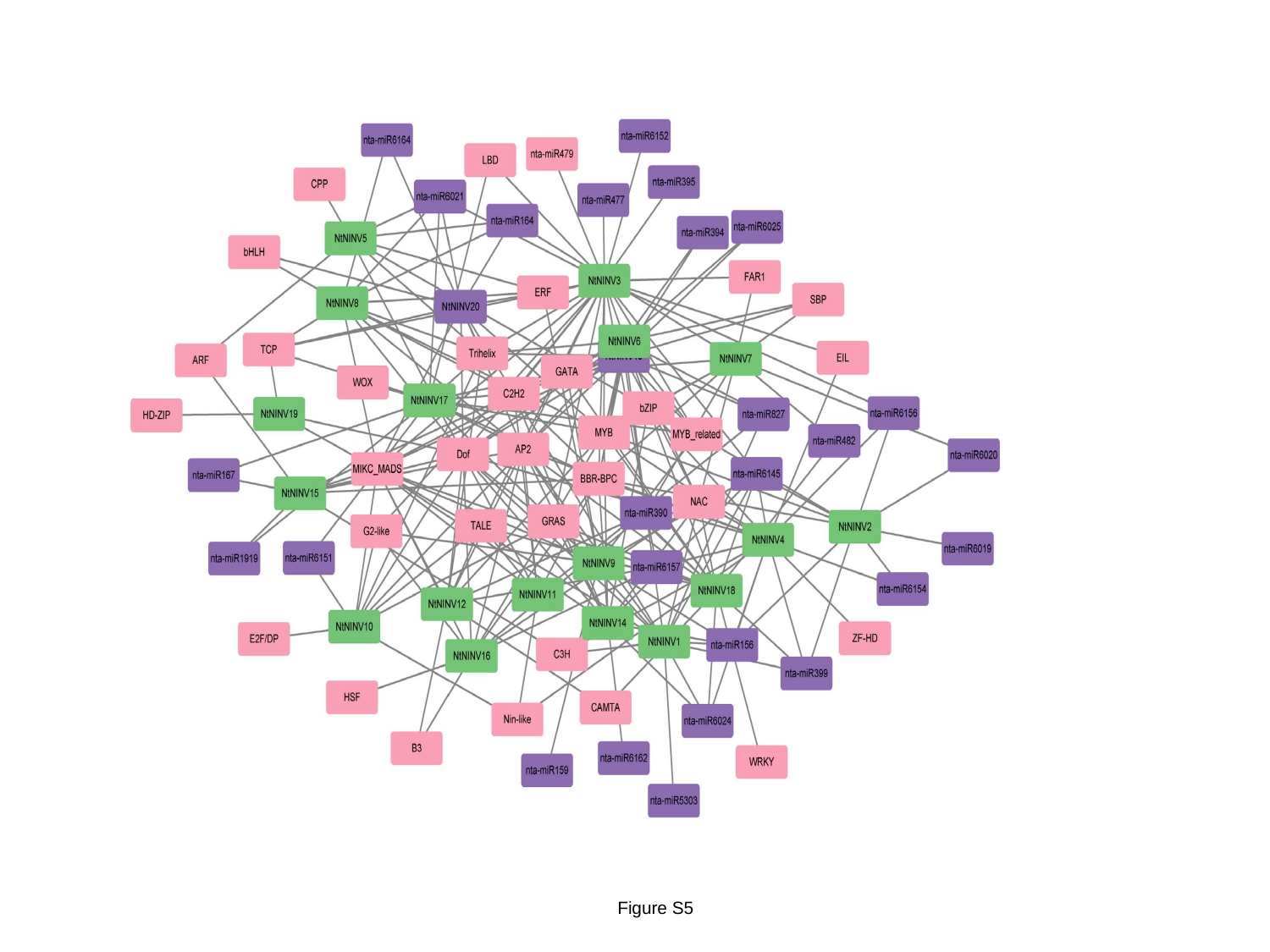

Figure S5

## Slide 6
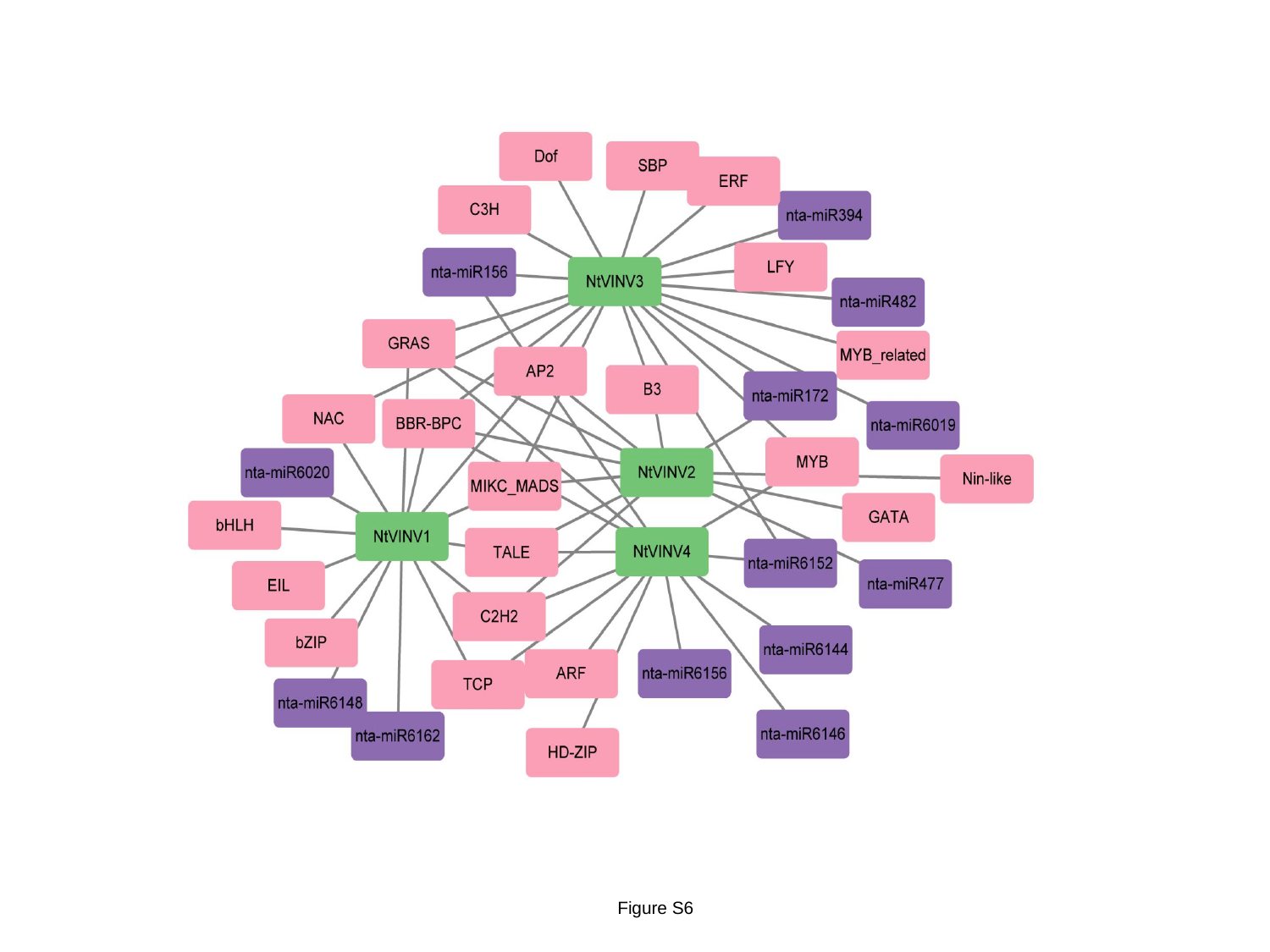

Figure S6

## Slide 7
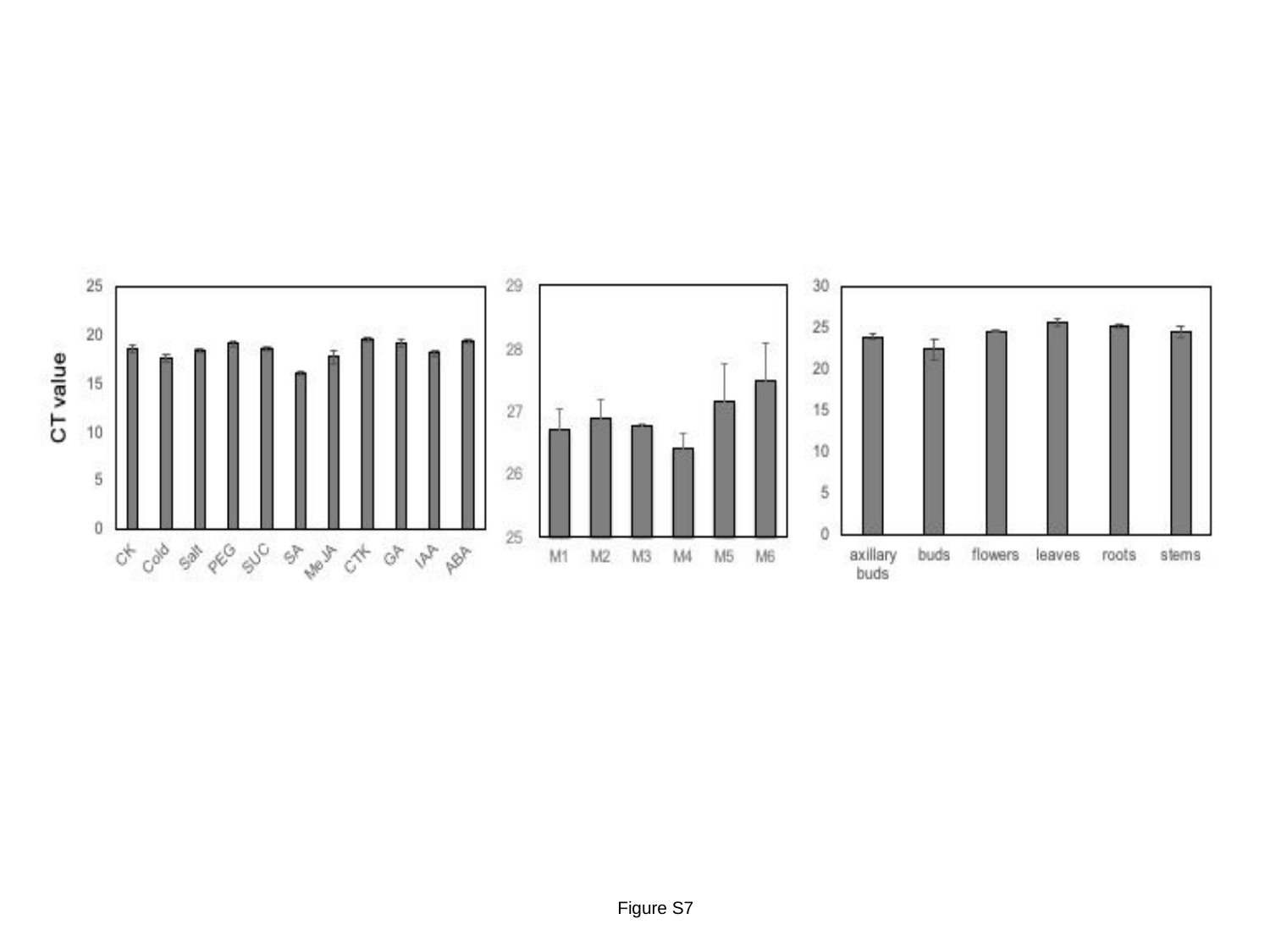

Figure S7

## Slide 8
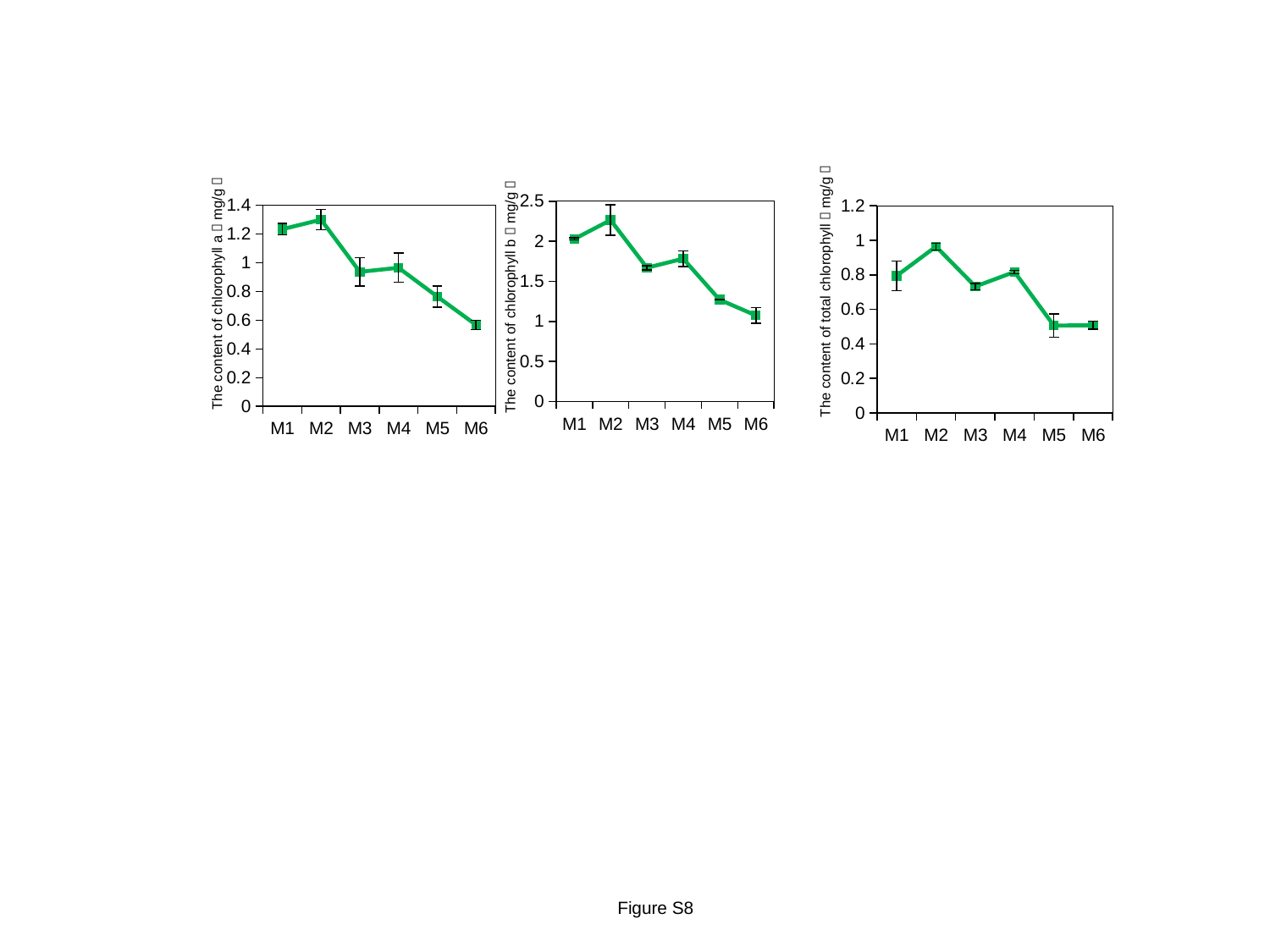

### Chart
| Category | |
|---|---|
| M1 | 1.2338144236760133 |
| M2 | 1.3000859567901235 |
| M3 | 0.93661380952381 |
| M4 | 0.96514177777778 |
| M5 | 0.7650156043956043 |
| M6 | 0.567316417445483 |
### Chart
| Category | |
|---|---|
| M1 | 2.0284704672897202 |
| M2 | 2.2639367901234566 |
| M3 | 1.6690312698412704 |
| M4 | 1.7819724444444467 |
| M5 | 1.2711872527472527 |
| M6 | 1.0747689408099688 |
### Chart
| Category | |
|---|---|
| M1 | 0.794656043613707 |
| M2 | 0.963850833333333 |
| M3 | 0.7324174603174604 |
| M4 | 0.8168306666666667 |
| M5 | 0.5061716483516484 |
| M6 | 0.5074525233644857 |The content of chlorophyll a（mg/g）
The content of chlorophyll b（mg/g）
The content of total chlorophyll（mg/g）
Figure S8

## Slide 9
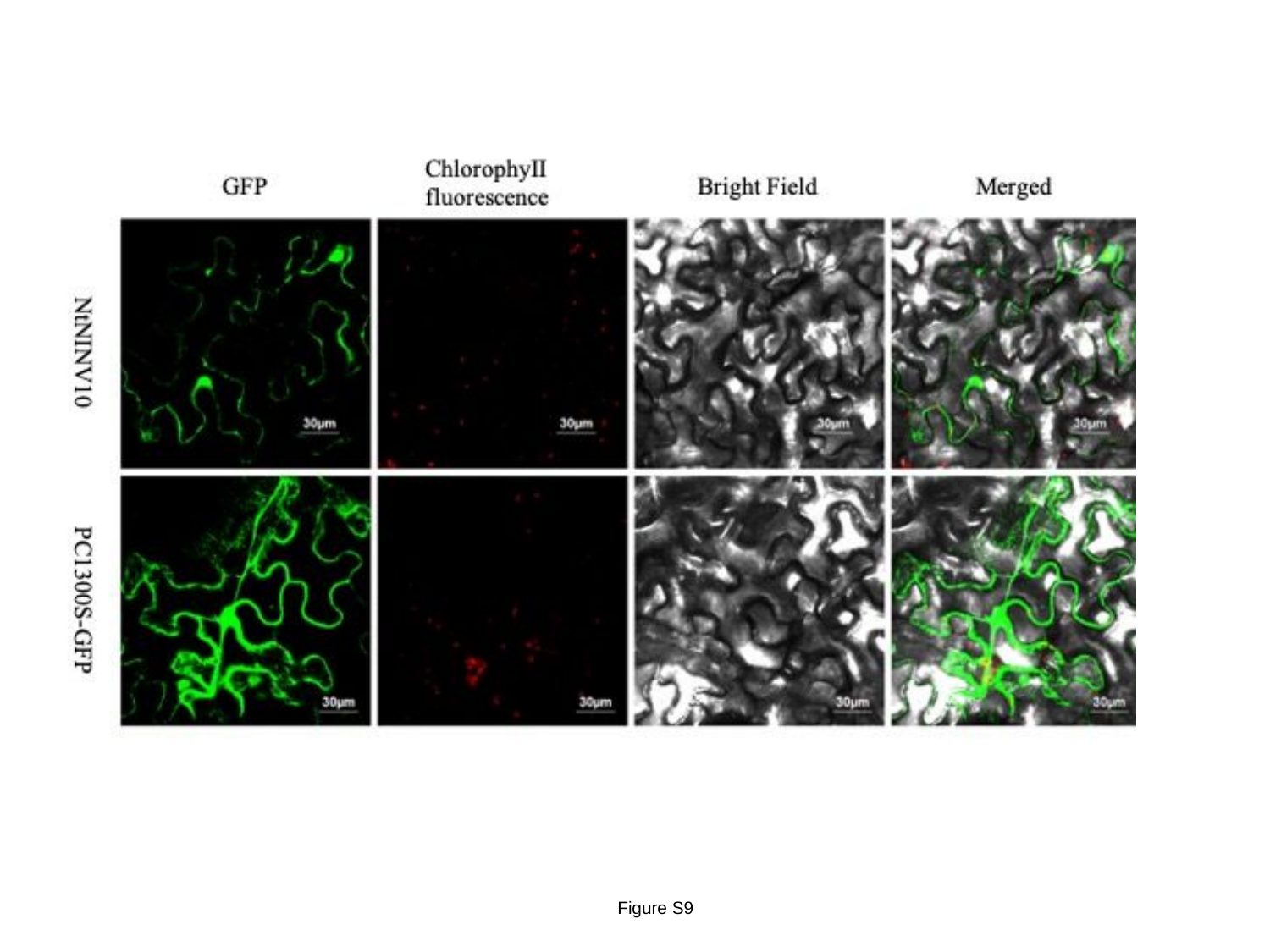

Figure S9

## Slide 10
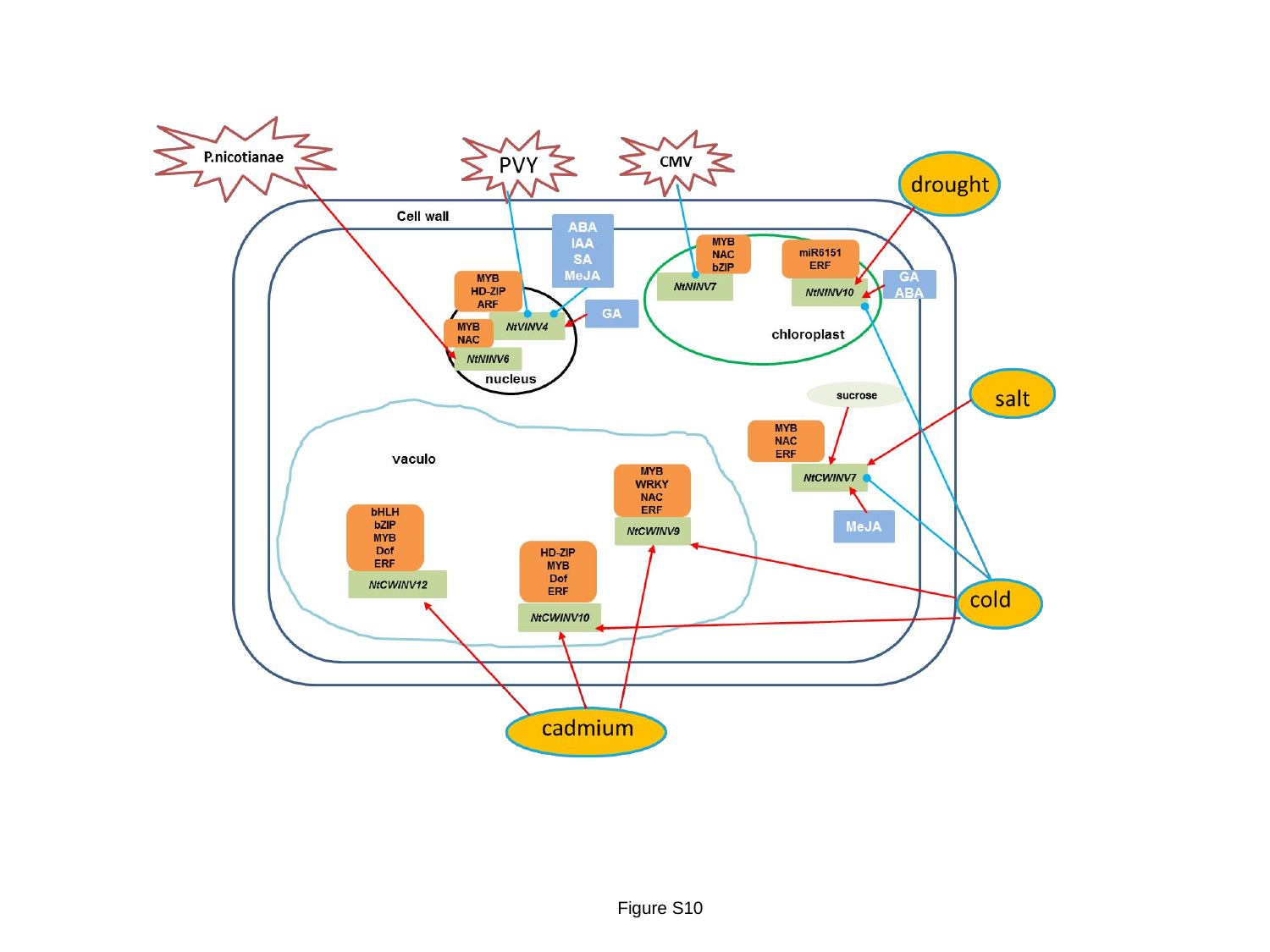

Figure S10
